# Scientific Items

**Published:** 1879-10-20

**Authors:** 


### SCIENTIFIC ITEMS.

**The Cause of Thunder.**—I have lately seen it stated in a text book upon electricity and magnetism that the phenomenon of thunder is not accounted for by any theory as yet brought forward. Whether this be so or not I am not sufficiently acquainted with the subject to say.

I believe that the commonly accepted theory is that a vacuum is created in the path of the electric spark, and that the subsequent inrush of the air produces the detonation. If, however, it be allowed that the electric spark is not a material substance, but merely a natural force or mode of motion, the possibility of this theory is at once disposed of. It is a well known fact that the passage of electrieity in a high state of tension, through a mixture of oxygen and hydrogen, not only causes an explosion, but also causes the formation of water, and it seems to me that, given the existence of free oxygen and hydrogen in the region of the electric disturbance, the phenomenon of thunder is sufficiently accounted for. Whether the normal amount of hydrogen in the air is sufficient to cause the great noise of thunder I am not competent to judge, but if not, I would suggest that the presence of an abnormal amount might be accounted for by the process of electrolysis, which would probably occur between the two poles of the thunder cloud before the tension became so great as to cause a rupture of the circuit and subsequent discharge of the electic spark. I would also draw your attention to the fact that every thunder clap is immediately followed by an increase in the quantity of water deposited in shape of rain; does not this point to the formation of water by the explosion of the gases? As I myself am unable, both from want of means and time, to investigate the matter, I should be glad to find that some one better qualified had taken the subject in hand. It is a frequent experiment of Dr. Tyndall's to show his audience red clouds, and I feel confident that by following this line of inquiry he could give us a real thunder storm.—*From Nature.*

**Solar Systems Other than Our Own.**—We know a great number of stars which are accompanied by smaller stars moving around them like the earth around the sun. These systems, which are now numbered by hundreds, have been so carefully observed that we have been able to calculate the orbits and periods of the planets, brilliant or opaque, which compose them.

It is then no longer on mere hypothesis that we can speak of solar systems other than our own, but with certainty, since we already know a great number, of every order and of every nature. Single stars should be considered as suns analagous to our own, surrounded by planetary worlds. Double stars, of which the second star is quite small, should be placed in the same class, for this second star may be an opaque planet reflecting only the light of the large one, or a planet still giving out heat and light. Double stars of which the two components give the same brightness are combinations of two suns. around each of which may gravitate planets invisible from this distance; these are worlds absolutely different from those of our system, for they are lighted up by two suns, sometimes simultaneous, sometimes successive, of different magnitudes, according to the distances of these planets from each of them; and they have double years of which the winter is warmed by a supplementary sun, and double days of which the nights are illuminated, not only by moons of different colors, but also by a new sun, a sun of night!

**Making the Deaf Hear.**—A new invention called the audiphone has recently been perfected. It is a simple affair, consisting of a fan of vulcanized rubber, slightly bent and held in tension by a silken cord, and placed in contact with the upper teeth. The thin rubber fan receives the vibrations and conveys them through the teeth.

Hon. Joseph Medill, of the Chicago Tribune, says of the invention in a recent number of that paper: "It is known that the editor of this paper has been deaf for a number of years, and that during that time he has used all the devices for improving his hearing, that he could hear of or that were brought to him. None of them were, however, satisfactory. He has tried the audiphone for some weeks, and finds that it not only improves his hearing, but restores the sense of hearing to him. Not merely does it answer when engaged in conversation with a person who is a foot, or a few feet from him, but it answers perfectly at a concert. Each note of the musician and each tone of the singer comes as clearly and distinctly as they did before the sense of hearing was impaired. Others have also tested this instrument, and have expressed themselves satisfied with its workings."—*Ex.*

**The Electric Light.**—Professor Cohn, of Breslau, has been lately making experiments with the electric light on the eyes of a number of persons for the purpose of testing its influence on visual perception and the sensation of color. He has found that letters, spots and colors are perceived at a much greater distance through the medium of electric light than by day or by gaslight. The sensation of yellow was increased sixty-fold, compared to daylight; of red six-fold; and of green and blue about two-fold. Eyes that could only with difficulty perceive and distinguish colors by daylight or gaslight were much aided by the electric light, and the visual perception was also much strengthened. Professor Cohn concludes from this fact that electric light would prove exceedingly useful in places where it is desirable that signals should be seen at a great distance. The engine used was Gramme's electro-magnetic apparatus, which rotates six hundred times in a minute.

**A Milk Test.**—A German paper gives a test for watered milk, which is simplicity itself. A well-polished knitting needle is dipped into a deep vessel of milk, and immediately withdrawn in an upright position. If the sample is pure, some of the fluid will hang to the needle; but if water has been added to the milk, even in small proportions, the fluid will not adhere to the needle.—*Cin. Med. News.*

THE discovery that oxides of nitrogen are given off by the voltaic arc is mentioned by Professor Tyndall as a source of injury in the use of the electric light. This discovery was recently made by Mr. Thomas Wills, of the Royal Naval College, an able young chemist, whose death is announced by the latest mails.

